# Multiplex PCR and Sequence Analysis to Investigate Genetic Diversity of *Fasciola* Isolates from Cattle and Sheep in Turkey

**DOI:** 10.3390/pathogens11111235

**Published:** 2022-10-26

**Authors:** Veysel Uzun, Figen Celik, Sami Simsek, Harun Kaya Kesik, Seyma Gunyakti Kilinc, Xiaocheng Zhang, Haroon Ahmed, Jianping Cao

**Affiliations:** 1 Department of Parasitology, Faculty of Veterinary Medicine, University of Firat, 23119 Elazig, Turkey; 2 Department of Parasitology, Faculty of Veterinary Medicine, Bingol University, 12000 Bingol, Turkey; 3 National Institute of Parasitic Diseases, Chinese Center for Disease Control and Prevention, (Chinese Center for Tropical Diseases Research), Shanghai 200025, China; 4 Key Laboratory of Parasite and Vector Biology, National Health Commission of the People’s Republic of China, Shanghai 200025, China; 5 World Health Organization Collaborating Center for Tropical Diseases, Shanghai 200025, China; 6 Department of Biosciences, COMSATS University Islamabad (CUI), Park Road, ChakhShazad, Islamabad 45550, Pakistan; 7 The School of Global Health, Chinese Center for Tropical Diseases Research, Shanghai Jiao Tong University School of Medicine, Shanghai 200240, China

**Keywords:** *Fasciola hepatica*, *F. gigantica*, *nad1*, haplotype, multiplex PCR, hybridization, introgression

## Abstract

Fasciolosis is a highly prevalent helminthic infection caused by *Fasciola hepatica* and *F. gigantica*. With the aim of identifying hybrid *Fasciola* flukes, multiplex PCR was performed to amplify the *pepck* gene. Furthermore, to determine *Fasciola* haplotypes, mitochondrial *nad1* gene was amplified and sequenced, and phylogenetic analyses were performed. Adult *Fasciola* isolates were collected from 51 cattle and 51 sheep, genomic DNA was isolated, and one-step multiplex PCR was subsequently performed to amplify *pepck*. Isolates that generated a 510 bp band were identified as *F. gigantica*, those that generated a 241 bp band were identified as *F. hepatica*, and those that generated both bands were identified as hybrid (aspermic) flukes. Multiplex PCR data identified four isolates as *F. gigantica* and 84 as *F. hepatica*. Fourteen hybrid isolates (five cattle and nine sheep) were identified. On unidirectional DNA sequence analysis of *nad1* PCR products, three sequences were identified as *F. gigantica* and 99 as *F. hepatica*. In addition, only 4 of 87 haplotypes detected for *F. hepatica nad1* sequences were found to be previously reported, while the remaining 83 are unique to this study. To summarize, this study is the first to report the existence of hybrid *Fasciola* flukes and 83 unique haplotypes of *F. hepatica* in Turkey.

## 1. Introduction

Fasciolosis is a parasitic liver infection mainly caused by *Fasciola* *hepatica* and *F. gigantica*, as well as by other digenetic trematodes in the Fasciolidae family [1]. According to The Centers for Disease Control and Prevention estimates, *F. hepatica* is prevalent in >70 countries across all continents, except Antarctica [2]. In a study focusing on the genetic characterization of *F. hepatica* in Argentina, 21 adult parasites were obtained from naturally infected bovine livers. Six haplotypes were detected with mitochondrial cytochrome c oxidase subunit 1 (mt-CO1), four haplotypes with mitochondrial NADH dehydrogenase subunit 4 (*nad4*), and three haplotypes with *nad5*, while no haplotypes were detected with ribosomal internal transcribed spacer 1 (ITS1) [3]. Furthermore, as per their analysis, the mt-CO1 gene fragment was the most variable marker. Another study reported the detection of 37 mitochondrial NADH dehydrogenase subunit 1 haplotypes in 130 *F. hepatica* isolates collected from abattoirs in nine provinces across Iran between 2015 and 2017 [4]. 

Both *F. hepatica* and *F. gigantica* reproduce bisexually via self-fertilization. They can be differentiated by their morphological characteristics [5]. These species show normal spermatogenic ability and are hermaphroditic [6]. Morphological and molecular studies have reported the existence of hybrid forms, known as aspermic intermediated forms [7]. Morphological hybrid forms produce offspring via interspecies fertilization and possibly reproduce parthenogenetically in Japan and other Asian countries [8,9,10,11,12]. Some reports have found that aspermic *Fasciola* spp. carry three different ITS1 genotypes [10]: Fh type, which is identical to *F. hepatica* sequences; Fg type, which is identical to *F. gigantica* sequences; and Fh/Fg type, which is a combination of both sequences. Aspermic *Fasciola* spp. show two major lineages of mitochondrial *nad1*. One belongs to *F. hepatica* and the other belongs to *F. gigantica*, indicating that these *Fasciola* forms originated from the same ancestors [11]. The presence of Fh/Fg type in ITS1, an instance of nuclear DNA, suggests that aspermic *Fasciola* forms are the result of a natural hybridization event between *F. hepatica* and *F. gigantica* [10,11,13]. The ITS1 region of ribosomal DNA shows hundreds of copies organized as tandem repeats. ITS1 analysis reportedly does not provide sufficient evidence of natural hybridization because repeat genes are highly recombinogenic and unstable, causing some controversies pertaining to *Fasciola* spp. characterization [14].

*Fasciola* isolates can be discriminated from one another on the basis of their nuclear protein-coding genes. Multiplex PCR methods have been optimized to differentiate *F. hepatica*, *F. gigantica*, and aspermic *Fasciola* flukes using the phosphoenolpyruvate carboxykinase (*pepck*) gene. Shoriki et al. [15] analyzed 27 *F. hepatica*, *F. gigantica*, and aspermic *Fasciola* flukes using nuclear (ITS1, *pepck*, and DNA polymerase delta (*pold*)) as well as mitochondrial (*nad1*) markers. They found that all aspermic *Fasciola* flukes displayed the Fh/Fg type in both *pepck* and *pold* regardless of their ITS1 genotypes and *nad1* lineages. In addition, a study characterized 196 *Fasciola* isolates in Spain as *F. hepatica* by multiplex PCR analysis of *pepck*, and 26 haplotypes were detected in mitochondrial *nad1*. It is notable that only one of them was previously reported in Spanish samples, indicating high genetic diversity and population structure in *F. hepatica* from Spain [16].

Giovanoli Evack et al. [17] collected flukes from *Fasciola*-positive cattle, sheep, and goats slaughtered in four Chadian abattoirs and extracted genomic DNA from 27 flukes. Subsequently, they performed species identification using the ITS1 + 2 locus. Of 27, 26 flukes were identified to be *F. gigantica*, while the remaining fluke showed heterozygosity at all variable sites that differentiate *F. hepatica* from *F. gigantica.* Upon cloning and sequencing of both alleles, the presence of one *F. hepatica* and one *F. gigantica* allele was confirmed. Their study was the first unambiguous molecular investigation to demonstrate the existence of such a hybrid in cattle in sub-Saharan Africa.

The present study was performed with a multiplex PCR aimed at amplifying *pepck* to identify hybrid forms of *Fasciola* isolates from cattle and sheep. The aim was to assess genetic diversity and haplotypes of *Fasciola* flukes by performing sequencing of mitochondrial *nad1*.

## 2. Materials and Methods

### 2.1. Collection of Adult Fasciola *spp.* Samples

Between February 2021 and February 2022, adult *Fasciola* spp. samples were collected from cattle and sheep from a slaughterhouse in Elazig province. Flukes from the liver of each animal were washed with distilled water, and bile residue was removed. All samples were stored in 70% ethanol at −20 °C until needed.

### 2.2. Genomic DNA Isolation 

The anterior one-third of the flukes’ bodies were excised into small pieces using a sterile scalpel and transferred to a 1.5 mL Eppendorf tube. The tissues were washed at least 4 to 5 times with 1 × PBS (pH = 7.4), and genomic DNA was then extracted using a Hibrigen Genomic DNA Isolation Kit (Hibrigen, Turkey). The isolated DNA was quantified with a Nanodrop spectrophotometer and stored at −20 °C until needed.

### 2.3. Amplification of Pepck by Multiplex PCR

To amplify *pepck*, a single-step multiplex PCR was performed using these primers: Fh-pepck-F, 5′-GATTGCACCGTTAGGTTAGC-3′; Fg-pepck-F, 5′-AAAGTTTCTATCCC GAACGAAG-3′; and Fcmn-Pepck-R, 5′-CGAAAATTATGGCATCAATGGG-3′ for *F. hepatica* and *F. gigantica*, respectively [15,18]. The 25 µL reaction mixture comprised 2.5 µL 10 × PCR buffer, 250 µM of each deoxynucleotide, 1.25 U Taq DNA polymerase, 20 pmol of each primer (0.4 µM Fh-pepck-F, 0.4 µM Fg-pepck-F, and 0.8 μM Fcmn-pepck-R), and 100 ng template DNA on average. The cycling conditions were as follows: initial denaturation at 94 °C for 1.5 min, followed by 30 cycles of denaturation at 94 °C for 30 s, hybridization at 61 °C for 30 s, and synthesis at 72 °C for 1 min, and then a final synthesis step at 72 °C for 10 min [15]. The amplicons thus obtained were separated on 1% agarose gel and bands were visualized with a UV transilluminator. Genomic DNA of isolates, which were previously confirmed to be *F. hepatica* and *F. gigantica* by sequencing [19], was included as positive control, and sterile distilled water served as negative control.

### 2.4. Amplification of nad1 by PCR

For exact species determination, genomic DNA of *Fasciola* isolates was amplified by PCR. Briefly, 5 μL 10 × PCR buffer, 250 μM of each deoxynucleotide, 1.25 U Taq DNA polymerase, 20 pmol of each primer to amplify *nad1* (Ita-10, 5′-AAGGATGTTGCTTTGTCGTGG-3′ and Ita-2, 5′-GGAGTACGGTTACATTCA-3′), and 100 ng template DNA on average were mixed to obtain a reaction mixture of 50 μL [11]. The cycling conditions were as follows: initial denaturation at 94 °C for 1.5 min, followed by 30 cycles of denaturation at 94 °C for 1.5 min, hybridization at 51 °C for 1.5 min, and synthesis at 72 °C for 2 min, and then a final synthesis step at 72 °C for 10 min. The amplicons thus obtained were subsequently subjected to 1.4% agarose gel electrophoresis, followed by ethidium bromide staining.

### 2.5. DNA Sequence Analysis

All *nad1* PCR products, obtained by amplifying genomic DNA of *Fasciola* spp. isolates with specific primers, were subjected to unidirectional DNA sequence analysis by a commercial company (BM Labosis, Ankara, Turkey); sequence ends were trimmed after they were compared with published sequence data using BLAST (http://www.ncbi.nlm.nih.gov/BLAST/). These trimmed sequences were then uploaded to MEGA X [20]. Alignment was performed using published reference sequences, and several other sequences from NCBI PubMed were added as outgroups. The most accurate evolutionary tree model was determined using the maximum likelihood method in MEGA X [20], and the tree was constructed with 1000 bootstrap values.

### 2.6. Haplotype Analysis

Sequencing data were exported to DnaSP 6 [21]. Population diversity indices (haplotype numbers (h), haplotype diversity (Hd), and nucleotide diversity (π)) and neutrality indices (Tajima’s D test [22], Fu’s statistics [23], and Fu and Li’s D and F tests [24]) were calculated using DnaSP 6 [21]. DnaSP 6 was used to create various output formats (e.g., NEXUS, which allows the user to add additional data for further analyses). A haplotype network was generated using PopART 1.7 (http://popart.otago.ac.nz) by applying the minimum spanning networks method, which contains all edges appearing in a minimum spanning tree [25].

## 3. Results

### 3.1. Multiplex PCR Data

*pepck* was subjected to multiplex PCR analysis with specific primers. Samples that generated a 510 bp band were considered to be *F. gigantica*, those that generated a 241 bp band were considered to be *F. hepatica*, and samples that generated both bands were considered to be hybrid forms (aspermic flukes). Of 102 *Fasciola* spp. isolates, only 4 (2 cattle and 2 sheep) generated a 510 bp band, and 84 (44 cattle and 40 sheep) generated a 241 bp band. Consequently, they were identified as *F. gigantica* and *F. hepatica*, respectively. Finally, 14 (five cattle and nine sheep) of 102 *Fasciola* spp. isolates generated both bands, and they were thus identified as hybrid forms.

### 3.2. nad1 Sequence Analysis

**Figure 1:**
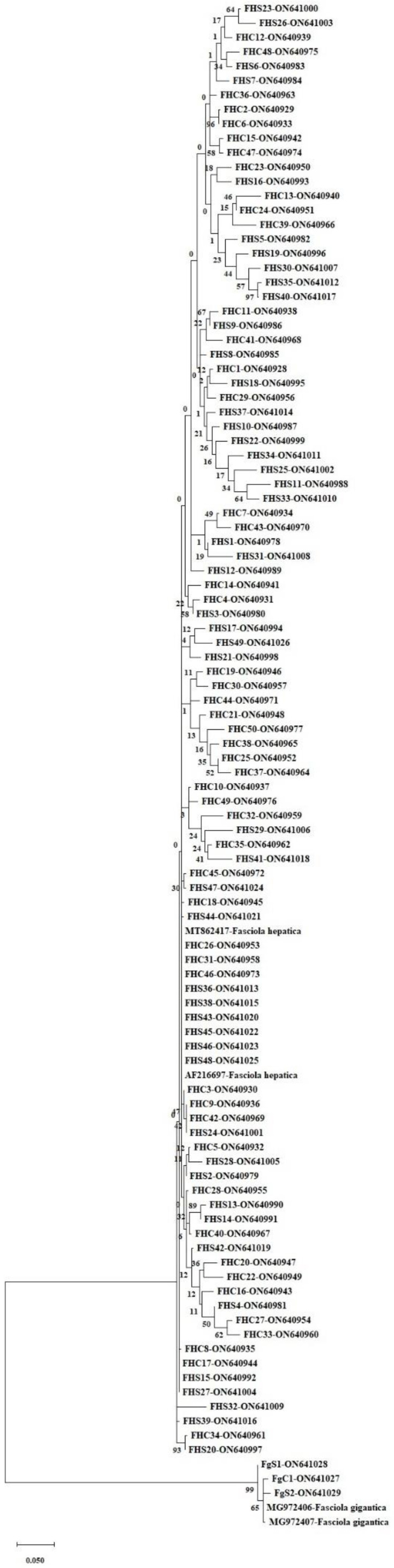
*Fasciola hepatica* phylogenetic tree constructed using *nad1* sequence of ON640928–ON640977 (FHC01-FHC50) and ON640978–ON641026 (FHS01-FHS49). *F. gigantica* phylogenetic tree constructed using nad1 sequences of ON641027 (FGC01), and ON641028-ON641029 (FGS01-FGS02) isolates. Reference sequences of *F. hepatica* were AF216697 and MT862417, and of *F. gigantica* were MG972406 and MG972407. The tree was created based on the GTR + G + I model with the maximum likelihood method in MEGA X, and reliability was assessed by performing 1000 bootstrap tests.

Unidirectional DNA sequence analysis of *nad1* (660 bp) of *Fasciola* spp. isolates (*n* = 102) was performed. Sequences were trimmed after being compared with published sequence data using BLAST, yielding sequences of 565 bp. Consequently, 3 sequences (encoding FGC01, FGS01, and FGS02), 2 from sheep and 1 from cattle, were identified as *F. gigantica* and 99 (FHC01–FHC50 and FHS01–FHS49), 50 from cattle and 49 from sheep, were identified as *F. hepatica*. All sequences were registered in GenBank. Figure 1 shows the phylogenetic tree constructed based on *F. gigantica* (*n* = 3) and *F. hepatica* (*n* = 99).

Table S1 shows the nuclear and mitochondrial DNA analysis findings of the flukes used in this study. 

### 3.3. Haplotype Analysis, Nucleotide Polymorphism, and Diversity and Neutrality Indices

**Figure 2:**
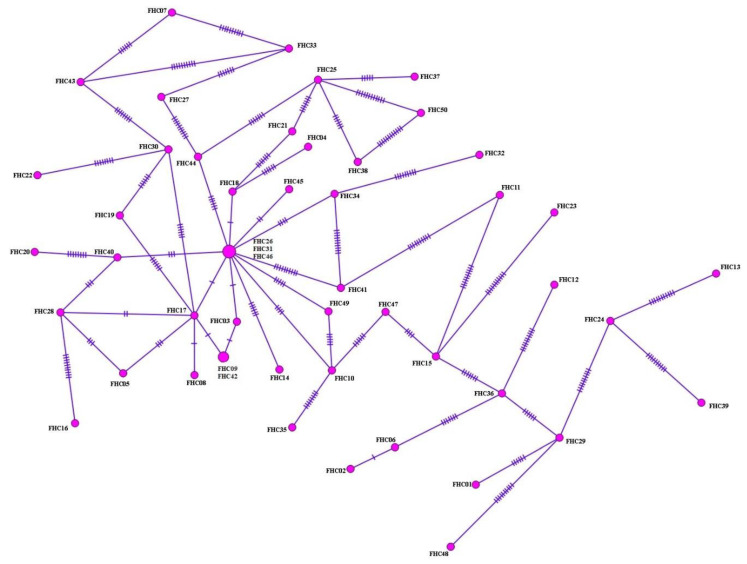
Haplotype network for nad1 of cattle isolates (565 bp) of *F. hepatica*. Circle size is proportional to the frequency of each haplotype. Numbers of mutations that distinguish haplotypes are indicated by dashes.

Haplotype analyses were performed on 50 samples identified as *F. hepatica* based upon *nad1* sequence analysis of cattle isolates. From the haplotype network, 47 haplotypes were identified, arranged in a star-like configuration with the main haplotype, separated from other haplotypes by 1–22 mutation steps. They covered 6% (3/50) of total isolates (Figure 2). In addition, with regard to *nad1* sequences, 68 polymorphic domains, 86.8% (59/68) of which were parsimony-informative sites, were detected. Additionally, high-haplotype and low-nucleotide diversities were observed (Table 1). Tajima’s D value was negative, expressing population expansion and/or selection purification. Significantly, negative Fu’s Fs values were obtained, indicating the presence of rare haplotypes, as would be expected from recent population expansion. Overall, 95.7% (45/47) of haplotype groups consisted of single haplotypes.

**Figure 3:**
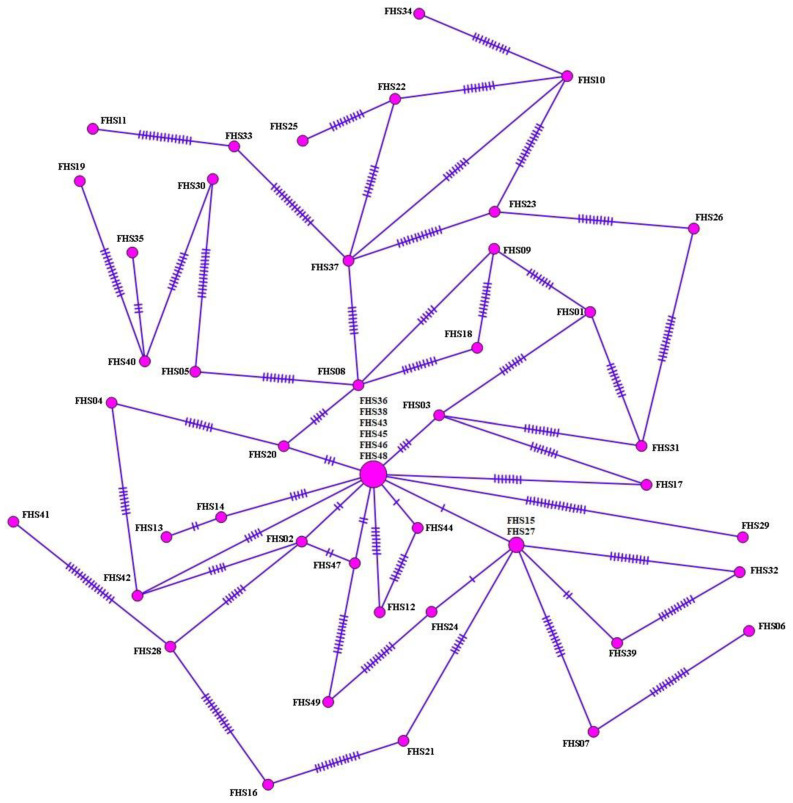
Haplotype network for *nad1* of sheep isolates (565 bp) of *F. hepatica*. Circle size is proportional to the frequency of each haplotype. Number of mutations that distinguish haplotypes is indicated by dashes.

Similarly, haplotype analysis was performed in 49 samples identified as *F. hepatica* based upon *nad1* sequence analysis of sheep isolates. From a haplotype network, 43 haplotypes were identified, arranged in a star-like configuration with the main haplotype, separated from other haplotypes by 1–26 mutation steps, and covering 12.2% (6/49) of total isolates (Figure 3). With regard to *nad1* sequences, 81 polymorphic domains were detected, 95.1% (77/81) of which were parsimony-informative sites. High-haplotype and low-nucleotide diversities were observed. Tajima’s D value was negative, expressing population expansion and/or selection purification. Significantly negative Fu’s Fs values were obtained, indicating the presence of rare haplotypes as expected from recent population expansion (Table 2). In total, 95.3% (41/43) of haplotype groups consisted of single haplotypes.

**Figure 4:**
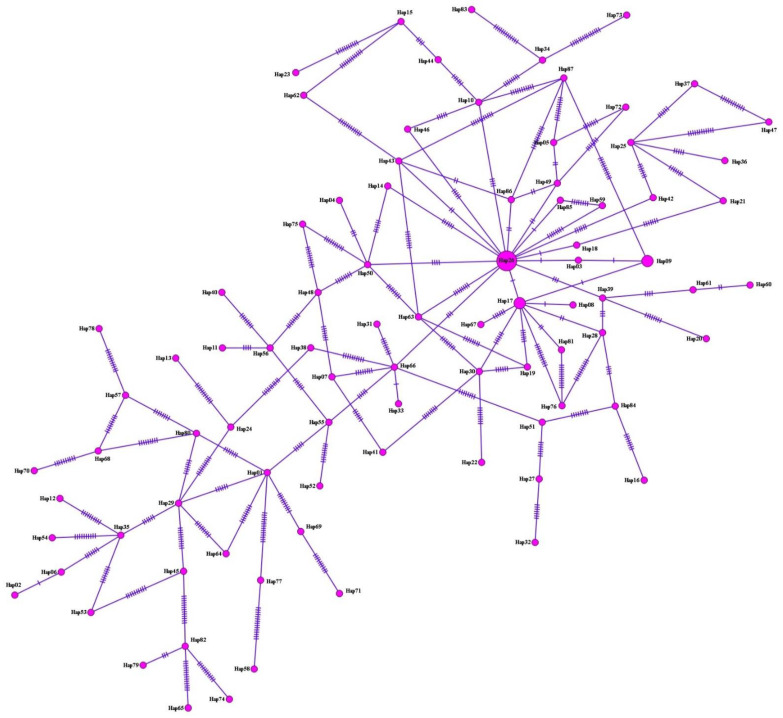
Haplotype network for *nad1* of both cattle and sheep isolates (565 bp) of *F. hepatica*. Circle size is proportional to the frequency of each haplotype. Number of mutations that distinguish haplotypes is indicated by dashes.

With regard to *F. hepatica*, on performing haplotype analysis of both cattle and sheep isolates (*n* = 99) for *nad1*, a common main haplotype was identified, separated from other haplotypes by 1–26 mutation steps. Overall, 87 haplotypes were detected, covering 9.1% (9/99) of total isolates (Figure 4, Table 3). For *nad1* sequences, 97 polymorphic domains were found, 92.8% (90/97) of which were parsimony-informative sites. High-haplotype and low-nucleotide diversities were observed. Tajima’s D value was negative, expressing population expansion and/or selection purification. Fu’s Fs values were significantly negative, indicating the presence of rare haplotypes, as expected from recent population expansion (Table 4). Additionally, 96.5% (84/87) of haplotype groups consisted of single haplotypes.

Of the 87 haplotypes detected for *F. hepatica nad1* sequences (*n* = 99), only 4 have been previously reported by other researchers, while the remaining 83 have been reported for the first time in the current study. Hap26, a previously identified haplotype, was the main haplotype in this study. Nine sequences of this haplotype showed a 100% match with a previously registered *F. hepatica* sequence (AF216697) in GenBank. Hap17, another previously identified haplotype, was the second main haplotype in this study. Although the sequences of this haplotype showed a 100% match with a previously reported *F. hepatica* sequence (MK468850), they were different from Hap26 by a single nucleotide. Similarly, Hap18 (FHC18) sequence was 100% identical to MG972379, but it was different from Hap26 by one nucleotide. Hap66 (FHS20) sequences were 100% identical to MN594514 and differed from Hap26 by three nucleotides. The 83 haplotypes identified for the first time in this study matched with *F. hepatica* sequences in GenBank at rates ranging from 96.11% to 99.82%. Although Hap03 (FHC03) and Hap85 (FHS44) sequences showed a 99.82% match with *F. hepatica* sequences, a single nucleotide was different between them and Hap26. Finally, the Hap58 (FHS11) sequence, which differed from Hap26 by as many as 26 nucleotides, was 96.11% identical to *F. hepatica* sequences.

Table S2 shows nucleotide variation positions of *nad1* (565 bp) among the 87 haplotypes detected in this study.

## 4. Discussion

Fasciolosis is a major and highly prevalent helminth infection, which is common in animals. The disease adversely affects the livestock sector, causing economic losses such as low fertility as well as meat, milk, and wool yield losses. Fasciolosis is mainly caused by *F. hepatica* and *F. gigantica*. *F. hepatica* is primarily distributed in the northern parts of Europe, America, Oceania, and Asia, while *F. gigantica* is mostly distributed in Africa and southern Asia [1]. In addition to these more recognized species, hybrid *Fasciola* flukes, resulting from cross-fertilization, have been found to be distributed in East, Southeast, and South Asian countries [10,11,12,26,27,28,29,30,31,32,33,34]. A few previous studies [19,34] have reported the differentiation of *Fasciola* isolates by molecular and morphometric analyses; however, these methods remain inadequate for the identification of hybrid species.

Ref. [19] performed PCR–RFLP analysis of partial mt-CO1 fragments using Turkish cattle isolates of *F. hepatica* (*n* = 16) and *F. gigantica* (*n* = 1). After digesting mt-CO1 PCR product with *AluI*, the RFLP profile of *F. hepatica* revealed two fragments (approximately 330 and 110 bp); however, the PCR product belonging to *F. gigantica* was not excised. Similarly, *RsaI* digestion generated two fragments (approximately 190 and 280 bp) from *F. gigantica*, but none from *F. hepatica*. Sequence analysis and alignment results revealed 100% and 98% identity for *F. hepatica* and *F. gigantica* isolates, respectively. As the existence of hybrid forms was unknown at the time, their status was not revealed [19].

New nuclear markers, in particular *pepck* and *pold*, have been successfully used to differentiate among *F. hepatica*, *F. gigantica*, and hybrid *Fasciola* flukes [10,11,12,26,27,28,29,30,31,32,33,34]. In addition, mt-CO1 and *nad1* have been used to analyze intraspecies phylogenetic relationships among *Fasciola* spp. [11].

In a study, 92 *Fasciola* flukes collected from sheep in Kabul, Afghanistan were subjected to multiplex PCR. All were identified as *F. hepatica* based on *pepck* and *pold* screening. Although the *pepck* fragment pattern was unable to distinguish the species of seven *Fasciola* isolates, *pepck* nucleotide sequence data validated that they were *F. hepatica* [35]. Further, Tashiro et al. [36] analyzed 68 *Fasciola* isolates collected from high-plateau and steppe areas of Algeria using multiplex PCR and PCR–RFLP for *pepck* and *pold*, respectively. In their study, multiplex PCR identified 49 isolates as *F. hepatica*, one as *F. gigantica*, and 18 as hybrids.

Variations in *pepck* nucleotides can affect primer specificity, which may cause errors in fragment analysis of multiplex PCR products. Therefore, results must be validated by performing mt-CO1 or *nad1* sequence analysis [35]. In this study, *Fasciola* isolates were analyzed by multiplex PCR and *pepck*-specific primers, and *nad1* sequence analysis was performed to identify genetic differences.

In previous studies in Turkey, liver flukes (usually obtained after slaughter) were identified as *F. hepatica,* but species discrimination was not conducted [37,38,39,40,41]. A morphometric and molecular study of *Fasciola* spp. from ruminants was conducted in Iran, and found that 48 (88.9%) of 54 *Fasciola* isolates were *F. hepatica* and 4 (7.4%) were *F. gigantica* [42]. Further, another study investigated the genetic diversity of *Fasciola* spp. in Armenia, and on morphological identification and sequencing of *nad1*, 55 specimens were identified as *F. hepatica* and 7 as *F. gigantica* [43]. Furthermore, 4 of 87 haplotypes detected in the current study for *F. hepatica nad1* sequences (*n* = 99) have been previously reported, while 83 were revealed for the first time in this study. One of the four previously reported haplotypes was from Australia, one was from Armenia, and two were from Iran. Hap26 was the main haplotype in this study; this haplotype was obtained using whole-genome sequencing of an *F. hepatica* isolate from Australia. Nine sequences of this haplotype showed a 100% match with a previously registered *F. hepatica* sequence (AF216697). Hap17, another previously determined haplotype, was the second main haplotype, and its sequence showed 100% match with a previously reported sequence of *F. hepatica* (MK468850), which was isolated from Iranian cattle. Hap17 and Hap26 sequences differed by a single nucleotide. Additionally, Hap18 (FHC18) sequence showed a 100% match with MG972379, which was obtained from a sheep isolate of Armenian origin. Hap18 and Hap26 sequences also differed by a single nucleotide. Furthermore, Hap66 (FHS20) sequence showed a 100% match with MN594514, which was obtained from a sheep isolate of Iranian origin. Hap66 and Hap26 sequences differed by three nucleotides [44]. The 83 haplotypes detected for the first time in this study matched with *F. hepatica* sequences in GenBank at rates ranging from 96.11% to 99.82%. One of the reasons for the high number of haplotypes detected in Turkey could be natural selection, which may lead to genetic differences between species. Diverse biological effects, such as different intermediate host preferences, tolerance to low or high temperatures, and sensitivity of parasites to anthelmintic drugs, could be responsible for genetic differences, resulting in haplotype diversity.

## 5. Conclusions

To summarize, the present study’s analyses led to the identification of hybrid *Fasciola* flukes, as well as 83 different haplotypes of *F. hepatica,* for the first time in Turkey. In the case of countries such as Turkey, where pasture farming is common and populations of cattle and sheep are large, the existence of hybrid *F. gigantica* and *F. hepatica* flukes is a major concern. Their presence may lead to the emergence of drug-resistant parasites and development of diseases that are more difficult and expensive to control. Consequently, not only animal but also human health can be endangered. Therefore, the implementation and maintenance of prevention and control strategies in fascioliasis-endemic regions can become a challenge.

## Figures and Tables

**Table 1:** Diversity and neutrality indices obtained using nucleotide data of *nad1* (565 bp) of *F. hepatica* in cattle isolates.

*n*	*h*n	hd ± SD	*π*d ± SD	Tajima’s D	*p* Value	Fu’s Fs	*p* Value	FLD	*p* Value	FLF	*p* Value
50	47	0.997 ± 0.005	0.02423 ± 0.00159	−0.43557	*p* > 0.10	−35.537	0.000	0.76537	*p* > 0.10	0.38471	*p* > 0.10

*n:* Isolate number, *h*n: haplotype number; *h*d: haplotype diversity; *π*d: nucleotide diversity; SD: standard deviation; FLD: Fu and Li’s D * statistic test; FLF: Fu and Li’s F * statistic test. *p* value: statistically not significant (*p* > 0.10).

**Table 2:** Diversity and neutrality indices obtained using nucleotide data of *nad1* (565 bp) of *F. hepatica* sheep isolates.

*n*	*h*n	hd ± SD	*π*d ± SD	Tajima’s D	*p* Value	Fu’s Fs	*p* Value	FLD	*p* Value	FLF	*p* Value
49	43	0.986 ± 0.011	0.02810 ± 0.00220	−0.62751	*p* > 0.10	−22.700	0.000	1.23614	*p* > 0.10	0.65299	*p* > 0.10

*n:* Isolate number, *h*n: haplotype number; *h*d: haplotype diversity; *π*d: nucleotide diversity; SD: standard deviation; FLD: Fu and Li’s D * statistic test; FLF: Fu and Li’s F * statistic test. *p* value: statistically not significant (*p* > 0.10).

**Table 3:** Distribution of the haplotype network created for *nad1* of *F. hepatica* for both cattle and sheep isolates. Number of isolates and their codes are shown.

Number	Haplotype Name	Isolate No.	Isolate Codes (Accession No.)
1	Hap01	1	[FHC01-ON640928]
2	Hap02	1	[FHC02-ON640929]
3	Hap03	1	[FHC03-ON640930]
4	Hap04	1	[FHC04-ON640931]
5	Hap05	1	[FHC05-ON640932]
6	Hap06	1	[FHC06-ON640933]
7	Hap07	1	[FHC07-ON640934]
8	Hap08	1	[FHC08-ON640935]
9	Hap09	3	[FHC09-ON640936 FHC42-ON640969 FHS24-ON641001]
10	Hap10	1	[FHC10-ON640937]
11	Hap11	1	[FHC11-ON640938]
12	Hap12	1	[FHC12-ON640939]
13	Hap13	1	[FHC13-ON640940]
14	Hap14	1	[FHC14-ON640941]
15	Hap15	1	[FHC15-ON640942]
16	Hap16	1	[FHC16-ON640943]
17	Hap17	1	[FHC17-ON640944 FHS15-ON640992 FHS27-ON641004]
18	Hap18	1	[FHC18-ON640945]
19	Hap19	1	[FHC19-ON640946]
20	Hap20	1	[FHC20-ON640947]
21	Hap21	1	[FHC21-ON640948]
22	Hap22	1	[FHC22-ON640949]
23	Hap23	1	[FHC23-ON640950]
24	Hap24	1	[FHC24-ON640951]
25	Hap25	1	FHC25-ON640952]
26	Hap26	9	[FHC26-ON640953 FHC31-ON640958 FHC46-ON640973 FHS36-ON641013 FHS38-ON641015 FHS43-ON641020 FHS45-ON641022 FHS46-ON641023 FHS48-ON641025]
27	Hap27	1	[FHC27-ON640954]
28	Hap28	1	[FHC28-ON640955]
29	Hap29	1	[FHC29-ON640956]
30	Hap30	1	[FHC30-ON640957]
31	Hap31	1	[FHC32-ON640959]
32	Hap32	1	[FHC33-ON640960]
33	Hap33	1	[FHC34-ON640961]
34	Hap34	1	[FHC35-ON640962]
35	Hap35	1	[FHC36-ON640963]
36	Hap36	1	[FHC37-ON640964]
37	Hap37	1	[FHC38-ON640965]
38	Hap38	1	[FHC39-ON640966]
39	Hap39	1	[FHC40-ON640967]
40	Hap40	1	[FHC41-ON640968]
41	Hap41	1	[FHC43-ON640970]
42	Hap42	1	[FHC44-ON640971]
43	Hap43	1	[FHC45-ON640972]
44	Hap44	1	[FHC47-ON640974]
45	Hap45	1	[FHC48-ON640975]
46	Hap46	1	[FHC49-ON640976]
47	Hap47	1	[FHC50-ON640977]
48	Hap48	1	[FHS01-ON640978]
49	Hap49	1	[FHS02-ON640979]
50	Hap50	1	[FHS03-ON640980]
51	Hap51	1	[FHS04-ON640981]
52	Hap52	1	[FHS05-ON640982]
53	Hap53	1	[FHS06-ON640983]
54	Hap54	1	[FHS7-ON640984]
55	Hap55	1	[FHS8-ON640985]
56	Hap56	1	[FHS9-ON640986]
57	Hap57	1	[FHS10-ON640987]
58	Hap58	1	[FHS11-ON640988]
59	Hap59	1	[FHS12-ON640989]
60	Hap60	1	[FHS13-ON640990]
61	Hap61	1	[FHS14-ON640991]
62	Hap62	1	[FHS16-ON640993]
63	Hap63	1	[FHS17-ON640994]
64	Hap64	1	[FHS18-ON640995]
65	Hap65	1	[FHS19-ON640996]
66	Hap66	1	[FHS20-ON640997]
67	Hap67	1	[FHS21-ON640998]
68	Hap68	1	[FHS22-ON640999]
69	Hap69	1	[FHS23-ON641000]
70	Hap70	1	[FHS25-ON641002]
71	Hap71	1	[FHS26-ON641003]
72	Hap72	1	[FHS28-ON641005]
73	Hap73	1	[FHS29-ON641006]
74	Hap74	1	[FHS30-ON641007]
75	Hap75	1	[FHS31-ON641008]
76	Hap76	1	[FHS32-ON641009]
77	Hap77	1	[FHS33-ON641010]
78	Hap78	1	[FHS34-ON641011]
79	Hap79	1	[FHS35-ON641012]
80	Hap80	1	[FHS37-ON641014]
81	Hap81	1	[FHS39-ON641016]
82	Hap82	1	[FHS40-ON641017]
83	Hap83	1	[FHS41-ON641018]
84	Hap84	1	[FHS42-ON641019]
85	Hap85	1	[FHS44-ON641021]
86	Hap86	1	[FHS47-ON641024]
87	Hap87	1	[FHS49-ON641026]

**Table 4:** Diversity and neutrality indices obtained using nucleotide data of both cattle and sheep isolates *F. hepatica nad1* (565 bp).

*n*	hn	hd ± SD	*π*d ± SD	Tajima’s D	*p* Value	Fu’s Fs	*p* Value	FLD	*p* Value	FLF	*p* Value
99	87	0.991 ± 0.005	0.02661 ± 0.00148	−0.85844	*p* > 0.10	−33.441	0.000	1.00700	*p* > 0.10	0.26059	*p* > 0.10

*n:* Isolate number, *h*n: haplotype number; *h*d: haplotype diversity; *π*d: nucleotide diversity; SD: standard deviation; FLD: Fu and Li’s D * statistic test; FLF: Fu and Li’s F * statistic test. *p* value: statistically not significant (*p* > 0.10).

## Data Availability

Not applicable.

## Supplementary Materials

The following supporting information can be downloaded at: https://www.mdpi.com/article/10.3390/pathogens11111235/s1. Table S1. Profiles of *Fasciola* flukes; Table S2. Nucleotide variation positions of *nad1* (565 bp) sequences among the 87 haplotypes analyzed in this study.
