# Supplementary material for: Multiplex PCR and Sequence Analysis to Investigate Genetic Diversity of Fasciola Isolates from Cattle and Sheep in Turkey
Source: Pathogens. 2022 Oct 26;11(11):1235. doi: 10.3390/pathogens11111235 (PMC9692402; doi:10.3390/pathogens11111235)
Supplement: Supplementary file 1 [file pathogens-11-01235-s001.zip › Supplement Table S2.pdf]

Nucleotide position	20	36	46	56	72	78	102	119	120	125	131	143	147	151	174	186	189	195	204	206	208	209	210	215	219	225	227	228	232
AF216697 (Reference sequence)	T	G	T	T	T	A	G	A	G	G	G	T	T	T	G	G	A	A	G	T	G	T	G	T	G	T	G	T	T
Hap01	C					G																							
Hap02																													
Hap03																													
Hap04																													
Hap05																							A						
Hap06																													
Hap07	C	A																											
Hap08																							A						
Hap09																							A						
Hap10																													
Hap11	C	A																											
Hap12																							A				C		
Hap13																							A						
Hap14																													
Hap15																													
Hap16																							A						
Hap17																							A						
Hap18																													
Hap19																							A						
Hap20																							A						
Hap21																													
Hap22																							A						
Hap23																													
Hap24	C																												
Hap25																													
Hap26																													
Hap27	C					G																							
Hap28																							A						
Hap29																													
Hap30																							A						
Hap31	C					G	T			T	A				T	C			T										
Hap32	C	A																											
Hap33	C					G																							
Hap34							T			T	A	A		C					T										
Hap35																							A						
Hap36																													
Hap37																													
Hap38	C					G																							
Hap39																													
Hap40	C	A																											
Hap41	C	A																											

Hap42																													
Hap43																													
Hap44																													
Hap45																	G												
Hap46																C													
Hap47																													
Hap48	C	A																											
Hap49																													
Hap50																													
Hap51	C					G																							
Hap52	C					G				T																			
Hap53																							A						
Hap54										T	A												A						
Hap55	C					G				T																			
Hap56	C	A																											
Hap57							T					A		C													C		
Hap58	C	A	C	A	C			T		T	A	A		C	T			T			A			A					
Hap59	C																												
Hap60																													
Hap61																													
Hap62																													
Hap63																													
Hap64	C					G																							
Hap65	C	C																											
Hap66	C					G																							
Hap67																							A						
Hap68												A						T						A					
Hap69																									A		C		
Hap70							T	T	C	T	A	A	A					T						A					A
Hap71										T		A													A	C	C		
Hap72															T								A						
Hap73	C	C	C	A			T	T	C	T	A	A		C						A				A					
Hap74	C																												
Hap75							T														A			A					
Hap76												A						T					A		A	C			
Hap77	C	A	C	A	C		T				A		A	C	T					A				A				A	
Hap78			C							T	A	A		C								C	A				C		
Hap79												A																	
Hap80															T													A	A
Hap81																							A						A
Hap82																													
Hap83							T			T	A	A		C															
Hap84																													
Hap85																													
Hap86																													
Hap87														C									A						

**Nucleotide position**	**235**	**245**	**253**	**254**	**255**	**265**	**273**	**282**	**291**	**296**	**301**	**303**	**304**	**309**	**317**	**320**	**321**	**325**	**334**	**337**	**343**	**346**	**348**	**349**	**351**	**354**	**357**	**363**	**365**
AF216697 (Reference sequence)	T	G	T	T	G	T	T	T	T	T	T	C	G	T	G	T	T	T	T	T	G	G	T	A	T	T	G	T	A
Hap01												T								C		T		T		A			
Hap02											C			C						C	C	T	C	T	A	A			
Hap03																													
Hap04															T					C		T		T					
Hap05													C																
Hap06											C			C						C	C	T	C	T	A	A			
Hap07			C									T																	
Hap08																													
Hap09																													
Hap10						C					C		C	C															
Hap11			C									T		C	T			C		C	C	T	C	T					
Hap12						C					C		C	C						C	C			T		A	T		
Hap13						C							C	C	T					C	C	T		T		A	T		
Hap14																			G					T					T
Hap15													C	C	T	G			G	C	C	T		T					
Hap16													C	C						C	C								
Hap17																													
Hap18																						T							
Hap19															T														
Hap20																													
Hap21																					C	T							
Hap22															T							T						C	T
Hap23													C	C	T					C	C	T				A			
Hap24												T		C						C	C	T		T		A			
Hap25																						T							
Hap26																													
Hap27												T																	
Hap28																													
Hap29												T								C	C	T		T		A			
Hap30															T													C	
Hap31									A			T		C															
Hap32			C									T																	
Hap33												T																	
Hap34						C		A	A		C		C	C															
Hap35														C						C	C	T		T		A	T		
Hap36																						T				A		C	T
Hap37																				C	C	T				A			
Hap38												T																	
Hap39																													
Hap40			C									T			T					C						A		C	T
Hap41			C									T			T														

Hap42																													
Hap43																					C								
Hap44											C		C	C		G			G		C			T					
Hap45											C		C	C				C		C	C	T	C	T					
Hap46											C			C											A				
Hap47													C							C	C	T							
Hap48			C									T												T		A			
Hap49																													
Hap50															T									T					
Hap51												T																	
Hap52												T		C						C	C					A			
Hap53		T				C								C				C		C	C	T		T		A			
Hap54						C					C									C	C	T		T					
Hap55												T								C		T		T		A			
Hap56			C									T								C	C	T	C	T		A			
Hap57										C		T								C		T		T		A			
Hap58		T				C	A		A		C	T								C		T		T		A			
Hap59																								T		A		C	
Hap60															T														
Hap61															T														
Hap62														C	T		C			C	C	T						C	
Hap63															T						C							C	
Hap64												T		C						C	C	T	C	T			C		
Hap65			C									T			T					C	C			T		A		C	
Hap66												T																	
Hap67															T						C					A	T	C	
Hap68	A	T			C							T								C		T		T		A			
Hap69						C	A	A			C		C							C		T		T		A			
Hap70	A	T										T								C		T		T		A			
Hap71	C					C	A	A		C	C		C											T		A			
Hap72														C			C			C	C								
Hap73	A	T				C								C															
Hap74												T			T					C	C			T			C		
Hap75								A		C														T		A			
Hap76						C	A					T																	
Hap77		T										T								C		T		T		A			
Hap78				A	C							T								C		T		T		A			
Hap79													C	C	T			C		C	C								
Hap80												T								C		T		T		A			
Hap81												T																	
Hap82													C	C	T			C		C	C			T					
Hap83		T					A	A	A					C			C			C	C						C		
Hap84																					C								
Hap85																													
Hap86																													
Hap87						C							C	C							C							C	

Nucleotide position	366	367	368	369	372	376	377	380	382	386	391	393	394	399	400	401	402	403	404	414	416	420	425	428	432	437	439	446	448
AF216697 (Reference sequence)	T	A	G	G	G	A	G	T	G	G	T	T	T	T	A	A	G	T	T	T	G	T	G	C	T	G	T	G	T
Hap01				T		C	T															C							
Hap02																					C								
Hap03																													
Hap04												G		C															
Hap05																					C								
Hap06				T																	C								
Hap07									T			G										C	T	T					
Hap08																													
Hap09																													
Hap10																													
Hap11																													
Hap12				T			T	C			C											C	T						
Hap13				T		C	T	C	T			G		C	T	C	T	G											
Hap14									T	T		G																	
Hap15				T																									
Hap16							T									C							T					T	C
Hap17																													
Hap18																													
Hap19							T					G				C							T	T					
Hap20												G						G			C		T						
Hap21		G		T			T		T														T	T				T	G
Hap22																	T						T	T					
Hap23				T		C					C	G		C								C	T	T				T	C
Hap24				T			T		T		C	G		C	T		T	G											
Hap25							T		T						T	C	T	G					T	T					
Hap26																													
Hap27							T								T		T						T	T		T	A	T	C
Hap28																													
Hap29			C	T			T																						
Hap30															T		T						T	T					
Hap31																													
Hap32																	T						T	T		T	A	T	C
Hap33																													
Hap34																													
Hap35				T																									
Hap36							T	C	T						T		T	G					T	T					
Hap37							T		T	T		G			T	C	T						T	T					
Hap38				T			T		T	T	C	G		C	T	C	T												
Hap39																												T	
Hap40	A																												
Hap41												G			T		T	G					T	T					

Hap42							T										T						T	T		T			
Hap43																						C							
Hap44				T																									
Hap45			C	T		C	T				C																		
Hap46				T																									
Hap47			C				T	C							T	C				C		C	T	T					
Hap48				T								G								C		C							
Hap49																					C								
Hap50												G								C									
Hap51							T															C	T					T	C
Hap52			C				T				C		C						C										
Hap53				T		C	T				C									C									
Hap54				T						T										C		C							
Hap55																													
Hap56																													
Hap57				T																		C							
Hap58				T																		C							
Hap59	C					C																				T			
Hap60																										T		T	
Hap61																												T	
Hap62				T	A						C		C							C		C			G				
Hap63							T					G					T						C						
Hap64				T			T			T											C								
Hap65				T	A		T				C		C				T	G	C	C	C								
Hap66																													
Hap67																									G				
Hap68				T																		C							
Hap69				T		C	T															C							
Hap70				T		C	T															C							
Hap71				T																C		C							
Hap72					A																C								
Hap73																													
Hap74			C			C	T	C	T	T	C		C				T	G	C			C							
Hap75				T								G								C		C							
Hap76							T															C	T						
Hap77				T		C	T															C							
Hap78				T		C	T															C							
Hap79				T	A	C	T		T	T	C		C				T	G	C			C							
Hap80				T																		C							
Hap81																													
Hap82				T	A	C	T		T	T	C		C				T	G	C										
Hap83					A			C														C							
Hap84																													C
Hap85																													
Hap86																					C	C							
Hap87								C									T				C	C							

Nucleotide position	473	476	486	491	507	516	525	532	544	547
AF216697 (Reference sequence)	T	T	A	T	T	T	T	G	A	G
Hap01										
Hap02	C									
Hap03							C			
Hap04										
Hap05	C									
Hap06	C									
Hap07										
Hap08					G					
Hap09							C			
Hap10										
Hap11										
Hap12										
Hap13										
Hap14										
Hap15										
Hap16	C		T	C						
Hap17										
Hap18										
Hap19										
Hap20	G	G	T	C						
Hap21										
Hap22		G	T	C						
Hap23										
Hap24										
Hap25										
Hap26										
Hap27	C			C						
Hap28	C		T							
Hap29										
Hap30										
Hap31										
Hap32	C		T							
Hap33										C
Hap34										
Hap35										
Hap36										
Hap37										
Hap38										
Hap39	G		T							
Hap40										
Hap41										

Hap42		G								
Hap43										
Hap44										
Hap45										
Hap46										
Hap47				C	G					
Hap48										
Hap49	C									
Hap50										
Hap51	C		T	C						
Hap52										
Hap53										
Hap54										
Hap55										
Hap56										
Hap57										
Hap58										
Hap59						C				
Hap60			T	C				C	T	
Hap61			T					C	T	
Hap62										
Hap63				C						
Hap64										
Hap65										
Hap66										
Hap67										
Hap68										
Hap69										
Hap70										
Hap71										
Hap72	C									
Hap73										
Hap74										
Hap75										
Hap76	C									
Hap77										
Hap78										
Hap79										
Hap80										
Hap81										
Hap82										
Hap83										
Hap84	C		T	C						
Hap85						A				
Hap86										
Hap87				C			C			
